# Evidence of islet CADM1-mediated immune cell interactions during human type 1 diabetes

**DOI:** 10.1172/jci.insight.153136

**Published:** 2022-03-22

**Authors:** Chandan Sona, Yu-Te Yeh, Andreas Patsalos, Laszlo Halasz, Xin Yan, Natalia L. Kononenko, Laszlo Nagy, Matthew N. Poy

**Affiliations:** 1 Institute for Fundamental Biomedical Research, Johns Hopkins All Children’s Hospital, St. Petersburg, Florida, USA.; 2 Division of Endocrinology, Diabetes and Metabolism, Department of Medicine, Johns Hopkins University School of Medicine, Baltimore, Maryland, USA.; 3 Stem Cell and Biotherapy Technology Research Center, College of Life Science and Technology, Xinxiang Medical University, Xinxiang, China.; 4 CECAD Excellence Center & Center for Physiology and Pathophysiology, Faculty of Medicine and University Hospital Cologne, University of Cologne, Germany.

**Keywords:** Autoimmunity, Endocrinology, Autoimmune diseases, Cell migration/adhesion, Diabetes

## Abstract

**BACKGROUND:**

Pathophysiology of type 1 diabetes (T1D) is illustrated by pancreatic islet infiltration of inflammatory lymphocytes, including CD8^+^ T cells; however, the molecular factors mediating their recruitment remain unknown. We hypothesized that single-cell RNA-sequencing (scRNA-Seq) analysis of immune cell populations isolated from islets of NOD mice captured gene expression dynamics providing critical insight into autoimmune diabetes pathogenesis.

**METHODS:**

Pancreatic sections from human donors were investigated, including individuals with T1D, autoantibody-positive (aAb^+^) individuals, and individuals without diabetes who served as controls. IHC was performed to assess islet hormones and both novel and canonical immune cell markers that were identified from unbiased, state-of-the-art workflows after reanalyzing murine scRNA-Seq data sets.

**RESULTS:**

Computational workflows identified cell adhesion molecule 1–mediated (*Cadm1*-mediated) homotypic binding among the most important intercellular interactions among all cell clusters, as well as *Cadm1* enrichment in macrophages and DCs from pancreata of NOD mice. Immunostaining of human pancreata revealed an increased number of CADM1^+^glucagon^+^ cells adjacent to CD8^+^ T cells in sections from T1D and aAb^+^ donors compared with individuals without diabetes. Numbers of CADM1^+^CD68^+^ peri-islet myeloid cells adjacent to CD8^+^ T cells were also increased in pancreatic sections from both T1D and aAb^+^ donors compared with individuals without diabetes.

**CONCLUSION:**

Increased detection of CADM1^+^ cells adjacent to CD8^+^ T cells in pancreatic sections of individuals with T1D and those who were aAb^+^ validated workflows and indicated CADM1-mediated intercellular contact may facilitate islet infiltration of cytotoxic T lymphocytes and serve as a potential therapeutic target for preventing T1D pathogenesis.

**FUNDING:**

The Johns Hopkins All Children’s Foundation Institutional Research Grant Program, the National Natural Science Foundation of China (grant 82071326), and the Deutsche Forschungsgemeinschaft (grants 431549029–SFB1451, EXC2030–390661388, and 411422114-GRK2550).

## Graphical abstract

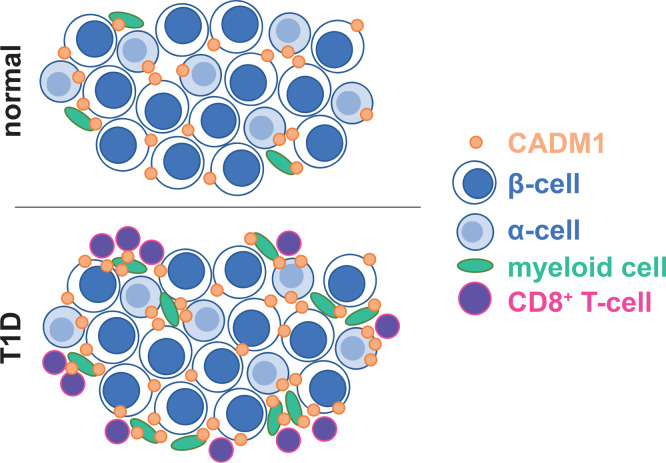

## Introduction

Type 1 diabetes (T1D) is characterized by hyperglycemia resulting from the autoimmune destruction of the insulin-expressing β cells of the pancreas (1–3). The pathology of T1D is illustrated by insulitis or the infiltration of inflammatory lymphocyte cells into the pancreatic islet, and CD8^^+^^ T cells are widely known to constitute an important component of the infiltrate (4, 5). The majority of these inflammatory cells are detected in the islet periphery and constitute the predominant lesion in the human pancreas (6–8). Important advances have been made in identifying islet cell molecules that are targeted during the autoimmune response during T1D, including the native protein and epitopes of proinsulin (9–13), islet-specific glucose-6-phosphatase catalytic subunit-related protein (14), GAD65 (15), insulinoma-associated antigen 2 (16), chromogranin (17), and islet-amyloid polypeptide (18). Despite this progress in understanding islet pathology during T1D (6, 7, 19), key conceptual gaps still remain in understanding the mechanisms that instigate immune cell infiltration and their recognition of β cells. Given the established role of autoreactive T cells in mediating β cell destruction, it is critical to emphasize the identification of islet proteins that mediate immune cell infiltration that could be targeted to both preserve pancreatic β cell mass and function and prevent the immune response that initiates T1D pathogenesis.

Cell adhesion molecule 1 (human CADM1 and mouse Cadm1) is an immunoglobulin-domain-containing membrane protein that mediates homotypic and heterotypic cell-to-cell contact with other Cadm family members and is expressed throughout the pancreatic islet, including pancreatic α and β cells as well as neuronal cells (20, 21). We showed previously that *Cadm1* (also referred to as *SynCAM1*, *Igsf4a*, *TSLC-1*, and *Necl2*) is directly targeted by miRNA 375 in the β cell and that suppression of *Cadm1* promotes insulin exocytosis and β cell mass (22–24). These results suggest that pharmacological inhibition of Cadm1 function could promote β cell secretion and growth, and its presence at the plasma membrane indicates multiple strategies may be developed to block its role in cell-to-cell contact.

In this study, we evaluated CADM1 expression in pancreatic sections from human donors with T1D to assess whether CADM1 intercellular interactions correlate with this disease state. We show that numbers of CADM1^^+^^ islet endocrine and myeloid cells adjacent to CD8^^+^^ T cells are increased in pancreatic sections from individuals with T1D and those who are autoantibody-positive (aAb^^+^^), compared with individuals who do not have diabetes (Non). In addition, single-cell sequencing analysis revealed enrichment of *Cadm1* expression in macrophages and DCs isolated from the pancreas of NOD mice, a model of autoimmune diabetes (25). Consistent with these observations, numbers of peri-islet CADM1^^+^^CD68^^+^^ myeloid cells adjacent to CD8^^+^^ T cells were also increased in pancreatic sections from individuals with T1D and those who were aAb^^+^^, compared with the Non group, indicating CADM1-mediated cell-cell contact may facilitate cytotoxic T lymphocyte (CTL) infiltration during autoimmune diabetes pathogenesis.

## Results

### Single-cell analysis identifies enrichment of Cadm1 expression in islet myeloid cell populations during the development of autoimmune diabetes in mice.

**Figure 1:**
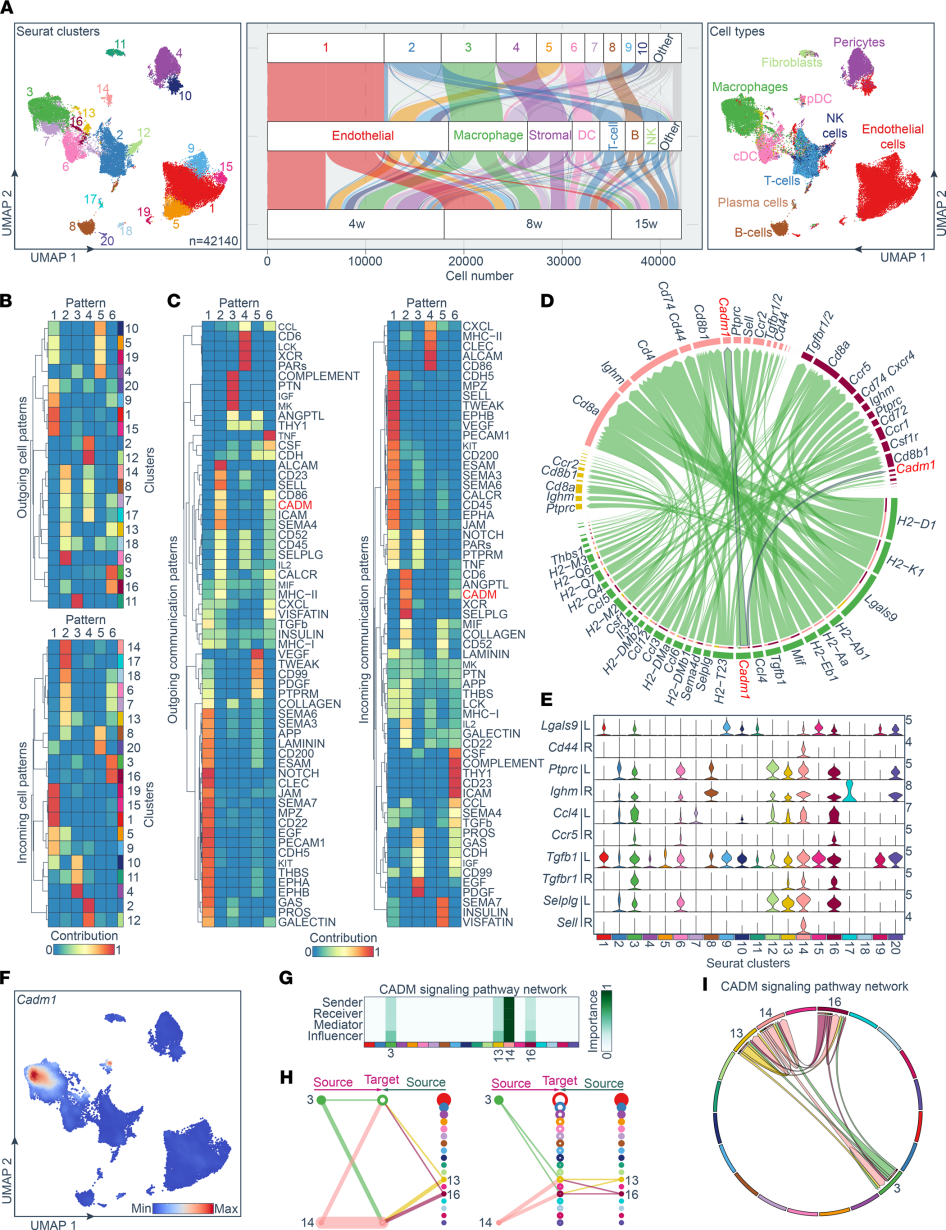
Evidence of Cadm1-mediated intercellular interaction within myeloid cell populations during autoimmune diabetes pathogenesis in mice. (**A**) UMAP and graph-based clustering of pancreatic islet immune cells from NOD mice between ages 4 and 15 weeks. The UMAP plot revealed cellular heterogeneity with 20 distinct clusters identified and color-coded. The general identity of each cluster was predicted by *SingleR* using the ImmGen database (right). The Sankey plot (middle) simultaneously defined the cellular identity for each cluster and time point. (**B**) Inferred outgoing (upper) and incoming (lower) communication patterns of secreting and target cells, which show correspondence between the inferred latent patterns and clusters and (**C**) the identified signaling pathways. (**D**) The chord diagram shows key macrophage communication pathways. The diagram links ligand-receptor pairs, which are grouped for cell type and cluster (colored outer arcs). Cadm1 is highlighted in red. (**E**) Violin plots showing distribution of established ligand-receptor gene pairs involved in the inferred signaling networks mediated by myeloid cells. (**F**) Feature plot of *Cadm1* distribution. Expression level for each cell are color-coded and overlaid onto a UMAP plot. (**G**) Heatmap shows the relative importance of cell groups based on the computed 4 network centrality measures of the CADM signaling network. (**H**) Hierarchical plot showing inferred intercellular communication network for CADM signaling. Left and right portions highlight autocrine and paracrine signaling between myeloid cell states, respectively. Solid and open circles represent source and target, respectively. Circle sizes are proportional to cell numbers in each group and edge width represents the communication probability. Edge colors are consistent with the signaling source. Cluster 3: macrophage; cluster 13: macrophage; cluster 14: DC; and cluster 16: macrophage. (**I**) Chord diagram shows CADM-signaling communication pathways used by different cell types. The links start from a ligand and end in a receptor, which are grouped for each cluster (colored outer arcs). pDC, plasmacytoid DC.

Single-cell RNA-sequencing (scRNA-Seq) analysis was performed on 42,140 total cells isolated from pancreatic islets from the NOD autoimmune mouse model at ages 4, 8, and 15 weeks to capture the progression of the disease (25). Leukocytes (CD45^^+^^) and islet vascular and mesenchymal cells were prepared using the 10x Genomics platform as described (25) and sequenced to capture changes in gene expression between the early and advanced prediabetic stages of the disease. Using a graph-based unsupervised clustering approach, filtering (Supplemental Figure 1, A–C; supplemental material available online with this article; https://doi.org/10.1172/jci.insight.153136DS1) and uniform manifold approximation and projection (UMAP) dimensionality reduction implemented within the Seurat package (R Foundation), 20 cell clusters were identified in the integrated data sets, including several immune cell types (T and B cells, macrophages, DCs, NK cells), endothelial cells, and mesenchymal cell populations (e.g., pericytes, fibroblasts) (Figure 1A and Supplemental Figure 1D). Cell identity assignments for each cluster were based on the expression of prominent marker genes (i.e., *Lyz2*, *Plac8*, *Ly6d*, *Lum*, *Cspg4*, *Emr1*, *H2-Oa*, *Cd3e*, *Klra1*, and *Pecam1*) using an automated, reference-based (established by the ImmGen database) scRNA-Seq annotation method (Figure 1A and Supplemental Figure 1E) (26, 27). One of the most abundant clusters, representing approximately 13.26% of all cells, was cluster C3. It was enriched for myeloid or macrophage markers, including *C1qa*, *Lyz2*, and *Emr1* (Supplemental Figure 1, E and F). Cluster C2 cells were classified as T lymphocytes (~13.78%), and they expressed *Cd3e*, *Nkg7*, and *Trbc2*, whereas cluster 12 was classified as NK cells by expressing *Klra1* (Supplemental Figure 1, E and F). Cluster C8 cells were identified as B lymphocytes (~4.29%) and cluster C6 as dendritic cells (~5.70%). Three other distinct cell clusters were C1 (~28.17%), C4 (~9.73%), and C11 (~1.43%). Cluster C1 cells were enriched for endothelial markers like *Pecam1*, cluster C4 cells were enriched for pericyte markers like *Cspg4*, and cluster C11 cells were collectively characterized as fibroblasts (Supplemental Figure 1, E and F). Cluster C11 cells were highly enriched for collagens, including Col3a1, and extracellular matrix protein genes, including *Dcn* and *Lum* (Supplemental Figure 1, E and F). Moreover, using differential expression testing, potentially novel markers can be extrapolated for each predicted cluster and cell type (Supplemental Figure 1C).

To begin to understand the functional crosstalk among the 20 identified cell clusters during diabetes pathogenesis, we used the computational workflow CellChat (http://www.cellchat.org/) to quantitatively infer and analyze intercellular communication networks from the scRNA-Seq data sets (28) (Figure 1B). To predict biologically relevant communications, CellChat identifies differentially overexpressed ligands and receptors for each cell group. The output of this analysis resulted in 2 sets of “communication patterns” that connect cell groups with signaling pathways either in the context of outgoing signaling (i.e., identifying cells as senders) or incoming signaling (i.e., identifying cells as receivers). Pattern 1 for outgoing signaling networks comprised an endothelial cell cluster (clusters 1, 5, 9, 10, 15, 19, and 20), including NOTCH, CLEC, EPHA, EPHB, KIT, EGF, and PECAM1 signaling pathways (Figure 1C). Pattern 2 integrated several immune cell types, including B cells, macrophages, and DCs (clusters 6–8, 13, 14, and 16–18) and included CADM, CD86, Sema4, IL2, MHCII, and Icam pathways. Pattern 6 similarly connected macrophage clusters 3, 13, and 16 and included CADM, MHCII, and TGF-β pathways, among others. Importantly, incoming signaling patterns indicated similar relevant pathways mediating known intercellular interactions and included CADM pathways in patterns 2 and 6, indicating the unbiased workflow captured the established role of Cadm1 proteins in mediating homotypic intercellular contact (Figure 1C) (20, 21).

By focusing on macrophage-mediated communication pathways, we identified several ligand-receptor pairs, including H2-Aa-Cd4, Lgals9-Cd44, Ptprc-Ighm, Ccl4-Ccr5, Tgfb-Tgfbr1, and Sell-Selplg (Figure 1D). Their gene expression predicts communication in previously shown cell types and subtypes, thus validating this analytical approach (Figure 1E). Moreover, *Cadm1*, a cell adhesion molecule mediating intercellular binding, was enriched in the macrophage and DC populations (Figure 1F). Notably, the CellChat workflow captured the inferred intercellular communication network for Cadm1 signaling and intercellular contact among the most important protein-protein interactions among all cell groups, with macrophages as a central influencer of the network (Figure 1, G–I).

**Figure 2:**
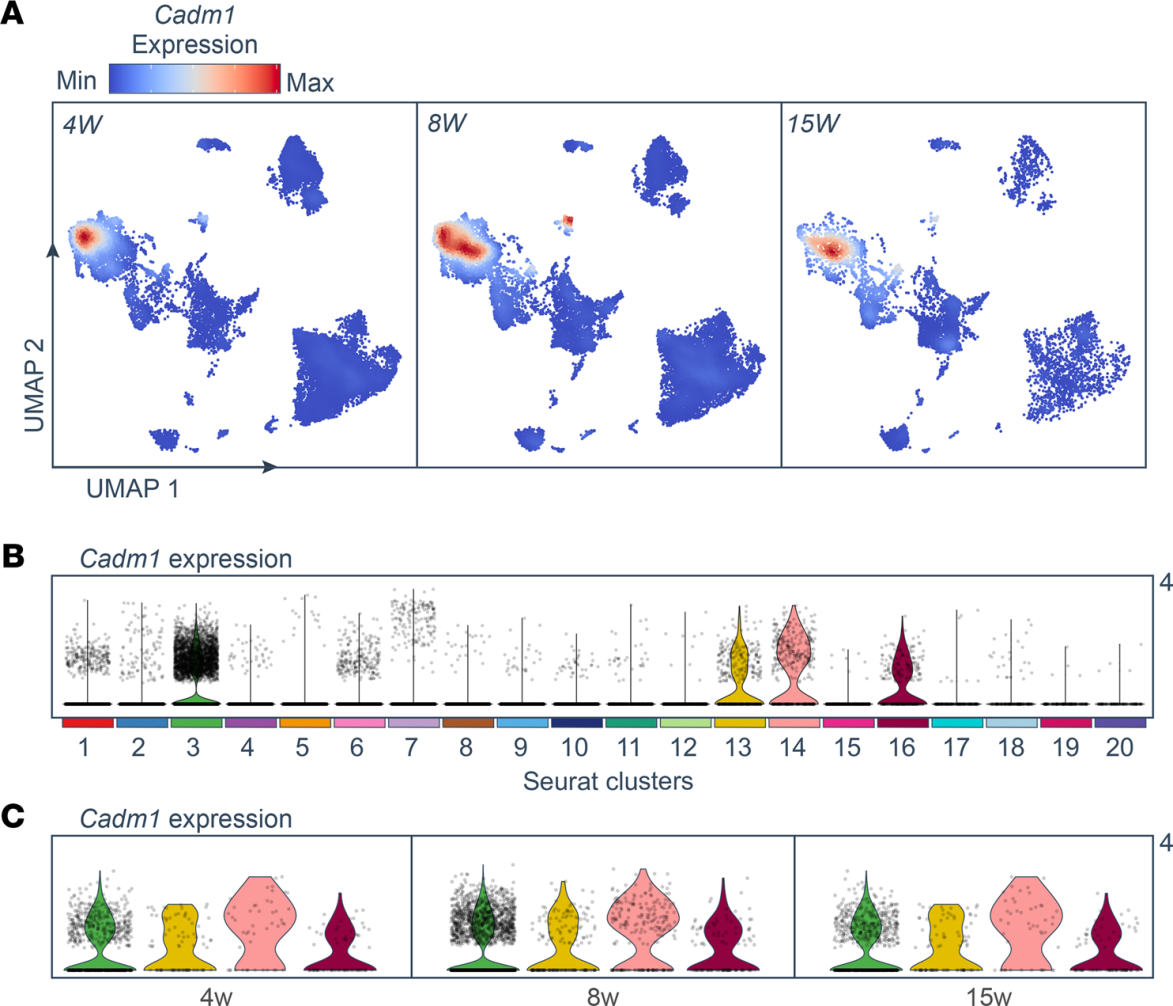
Increased number of Cadm1^+^ macrophages in pancreata of NOD mice at age 8 weeks. (**A**) Feature plot of expression distribution for Cadm1 per time point. Expression levels for each cell are color-coded and overlaid onto a UMAP plot. (**B**) Violin plots showing the expression distribution of Cadm1 in all identified clusters. Black dots indicate individual cells within each cluster. (**C**) Violin plots showing the expression of Cadm1 specifically in myeloid clusters per time point. All clusters follow the color code used in Figure 1A.

Temporal analysis of *Cadm1* expression across each time point showed that the number of *Cadm1*^^+^^ macrophages was highest at age 8 weeks, compared with at ages 4 and 15 weeks, and the highest number of *Cadm1*^^+^^ cells was within the macrophage group (cluster 3) (Figure 2, A and B). Interestingly, the number of *Cadm1*^^+^^ macrophages was lowest at age 15 weeks, which generally coincides with the manifestation of hyperglycemia in NOD mice and can indicate that Cadm1 function in intercellular binding may be relevant to diabetes pathogenesis around the age of 8 weeks in these animals (Figure 2C). Furthermore, the scRNA-Seq analysis revealed an alteration in the distribution of *Cadm1*^^+^^ macrophages at age 8 weeks compared with 4 weeks and may suggest the emergence of cellular heterogeneity within the macrophage cell cluster. Meanwhile, *Cadm1* expression followed a similar trend in conventional and plasmacytoid DCs (clusters 13 and 14, respectively), with the highest number of *Cadm1*^^+^^ cells in these groups at age 8 weeks (Figure 2C). The identification of Cadm1 as a potent mediator of intercellular contact among myeloid cells from the NOD model posed a novel hypothesis for an investigation into autoimmune diabetes pathogenesis, and we next sought to determine whether CADM1 expression in these immune cells contributes to direct interactions with CD8^^+^^ T cells within the islet microenvironment during human T1D.

### Increased number of CADM1^^+^^CD45^^+^^ cells within the pancreatic islet during T1D.

To test our hypothesis that CADM1 expression in islet cells contributes to immune cell infiltration during autoimmune diabetes in humans, we obtained paraffin-embedded pancreatic sections from the Non group, aAb^^+^^ individuals, and persons with T1D from the Network for Pancreatic Organ donors with Diabetes (nPOD) tissue repository at the University of Florida (Supplemental Table 1). Tissue sections from 5 independent donors from each group were obtained for immunostaining experiments (Supplemental Table 1). We first quantified β, α, and CD45^^+^^ cell numbers to validate the loss of insulin^^+^^ cells and the presence of insulitis in these samples (Supplemental Figure 2A). As expected, the number of islets with more than 15 CD45^^+^^ cells within the islet boundary (the definition of insulitis) was significantly higher in pancreata of individuals with T1D than in the other 2 groups (Supplemental Figure 2B) (29). Moreover, β cell numbers were diminished, whereas α cell number and CD45^^+^^ cell number were increased in pancreatic sections when comparing donors with T1D with aAb^^+^^ individuals and the Non control group (Supplemental Figure 2, C–F). The increased detection of CD45^^+^^ cells adjacent to or infiltrating the islet confirmed the increased presence of leukocyte cells throughout the exocrine pancreas and within the islets in pancreatic sections of our T1D cohort (Supplemental Figure 2, E–G) (30).

**Figure 3:**
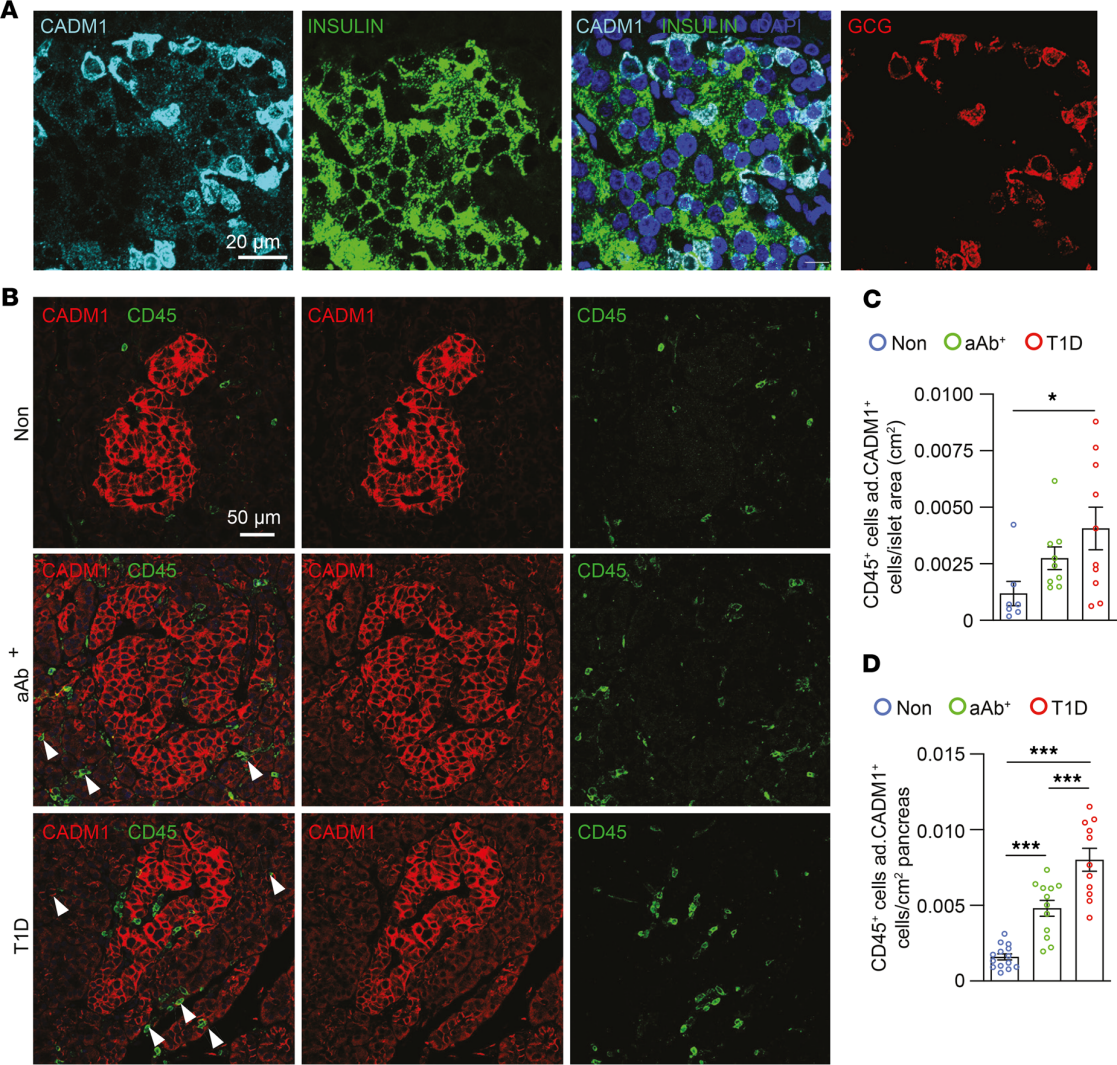
Increased number of CADM1^+^CD45^+^ cells within the pancreatic islet during T1D. (**A**) Immunostaining of paraffin-embedded pancreata from individuals in the Non group for CADM1 (cyan), insulin (green), and GCG (red). Scale bar: 20 μm. (**B**) Immunostaining of paraffin-embedded pancreata from individuals in the Non, aAb^+^, and T1D groups for CADM1 (red) and CD45 (green). Scale bar: 50 μm. (**C**) Quantification of the number of CADM1^+^CD45^+^ cells within the islet periphery (*n* = 5 per group). (**D**) Quantification of the number of CD45^+^ adjacent to CADM1^+^ cells per area pancreas (*n* = 5 per group). One-way ANOVA was performed using GraphPad Prism, version 7, software for comparisons of 3 groups. Post hoc statistical analyses were performed using the Tukey multiple comparisons test. Results are presented as mean ± SEM. *P < 0.05; ***P < 0.001. ad., adjacent.

Upon performing these control experiments, we then evaluated CADM1 and CD45 immunostaining in the pancreatic sections of each test group (i.e., Non, aAb^^+^^, and T1D) (Figure 3A). As expected, CADM1 was detected at the plasma membrane throughout the endocrine pancreas in sections from the Non group, and the number of CADM1^^+^^CD45^^+^^ cells was elevated in the aAb^^+^^ and T1D groups, indicating a significant number of islet leukocyte cells were CADM1^^+^^ (Figure 3, B–D) (21). This observation is consistent with the scRNA-Seq results from NOD mice identifying *Cadm1* expression in islet myeloid cell populations, potentially establishing a parallel between this established mouse model of autoimmune diabetes and the human form of the disease. Interestingly, CADM1 expression was sparsely detected in the exocrine pancreas of Non individuals, and its expression became widespread in the acinar cells of aAb^^+^^ individuals and those with T1D (Figure 3B). This observation may allude to a role for CADM1 expression in the exocrine pancreas in mediating immune cell infiltration of the pancreas during the pathogenesis of autoimmune diabetes. These results establish that there is increased detection of CADM1 in both CD45^^+^^ as well as acinar cells in pancreatic sections of aAb^^+^^ individuals and persons with T1D. In addition to CADM1 expression in islet endocrine cells, these observations also reveal multiple sites of potential interaction for infiltrating lymphocytes for binding CADM1-expressing islet cells.

### Increased number of CD68^^+^^ cells adjacent to CADM1^^+^^insulin^^+^^ cells during T1D.

**Figure 4:**
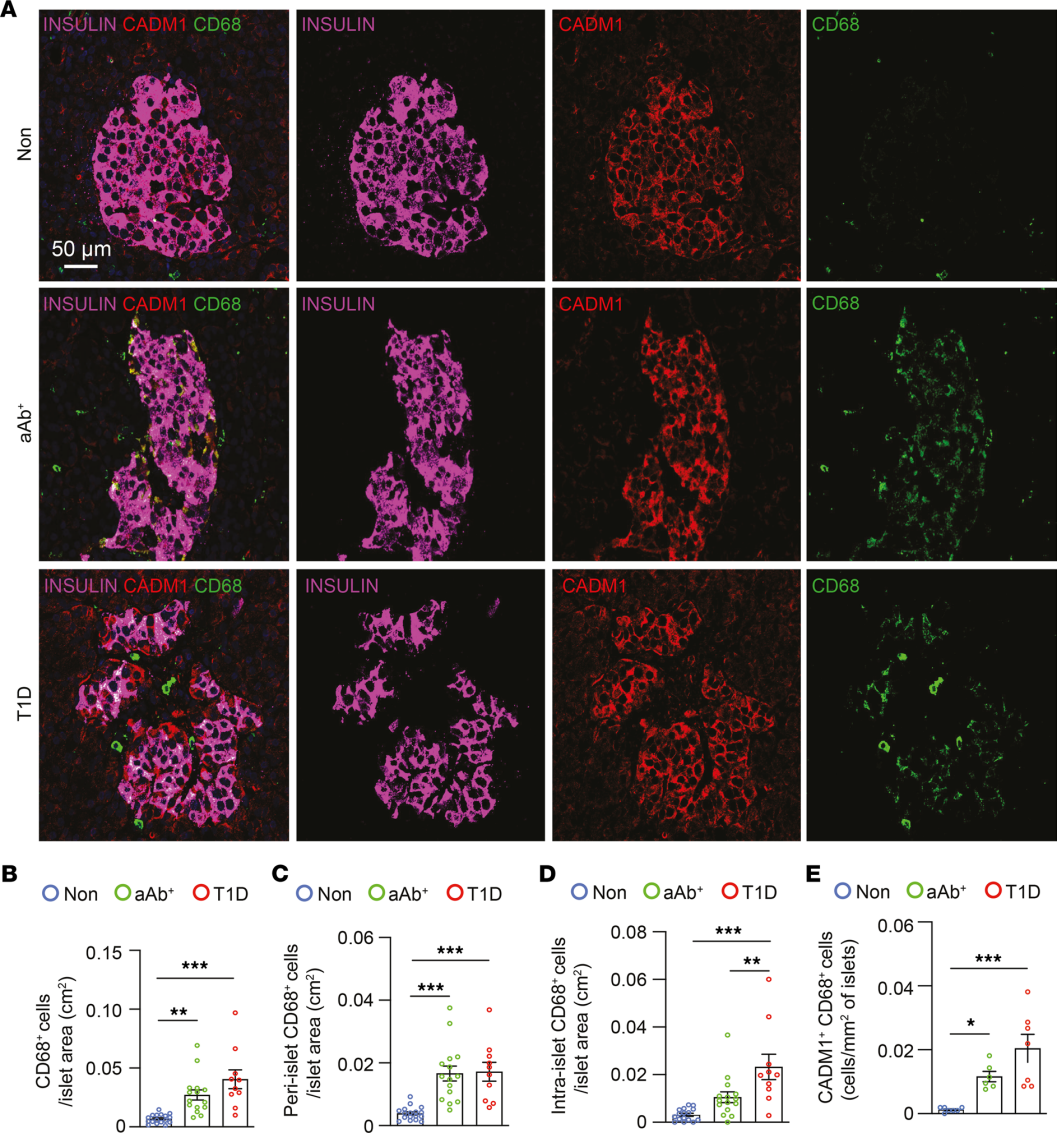
Increased number of CD68^+^ cells adjacent to CADM1^+^insulin^+^ cells during T1D. (**A**) Immunostaining of paraffin-embedded pancreata from individuals in the Non, aAb^+^, and T1D groups for CADM1 (red), CD68 (green), and insulin (magenta). Scale bar: 50 μm. (**B**) Quantification of the number of CD68^+^ cells within the islet boundary (*n* = 5 per group). (**C**) Quantification of the number of CD68^+^ cells at the islet periphery per islet area (*n* = 5 per group). (**D**) Quantification of the number of CD68^+^ cells within the islet boundary (*n* = 5 per group). (**E**) Quantification of the number of CADM1^+^CD68^+^ cells within the islet boundary (*n* = 5 per group). One-way ANOVA was performed using GraphPad Prism, version 7, software for comparisons of 3 groups. Post hoc statistical analyses were performed using Tukey’s multiple comparisons test. Results are presented as mean ± SEM. *P < 0.05; **P < 0.01; ***P < 0.001.

To test whether the increased number of CADM1^^+^^CD45^^+^^ cells within the islets of individuals with T1D were positive for macrophage markers, we next performed immunostaining for CD68 together with CADM1 and insulin. Similar to our observations of CD45^^+^^ cells, the number of CD68^^+^^ cells increased in pancreatic sections from the aAb^^+^^ and T1D groups compared with those from the Non group, and the majority of these cells were also CADM1^^+^^ (Figure 4, A and B). Furthermore, quantification of CD68^^+^^ cells at the islet periphery or within the islet also showed that CD68^^+^^ cell numbers were elevated in both the aAb^^+^^ and T1D groups (Figure 4, C and D), and CADM1^^+^^CD68^^+^^ cell numbers were similarly increased in these groups compared with the Non group (Figure 4E). Together, these results show (a) increased numbers of CADM1^^+^^ myeloid cells within the islets of aAb+ human donors and donors with T1D, and (b) strong colocalization of CADM1 with insulin adjacent to CADM1^^+^^ myeloid cells implies potential CADM1-mediated binding between immune and islet endocrine cell types.

Because CD68 is reported as a marker for human DCs, we also performed immunostaining for CD11c, a membrane protein present on DCs, neutrophils, and macrophages, and we observed increased numbers of CD11c^^+^^ cells in both aAb^^+^^ and T1D pancreata (Supplemental Figure 3, A–C). This increase in number of CD11c^^+^^ cells within islets of pancreatic sections from these 2 groups indicates that CADM1^^+^^ myeloid cells detected within the islet may be either mature macrophages or DCs.

### Increased number of CD8^^+^^ T cells adjacent to CADM1^^+^^ cells during T1D.

**Figure 5:**
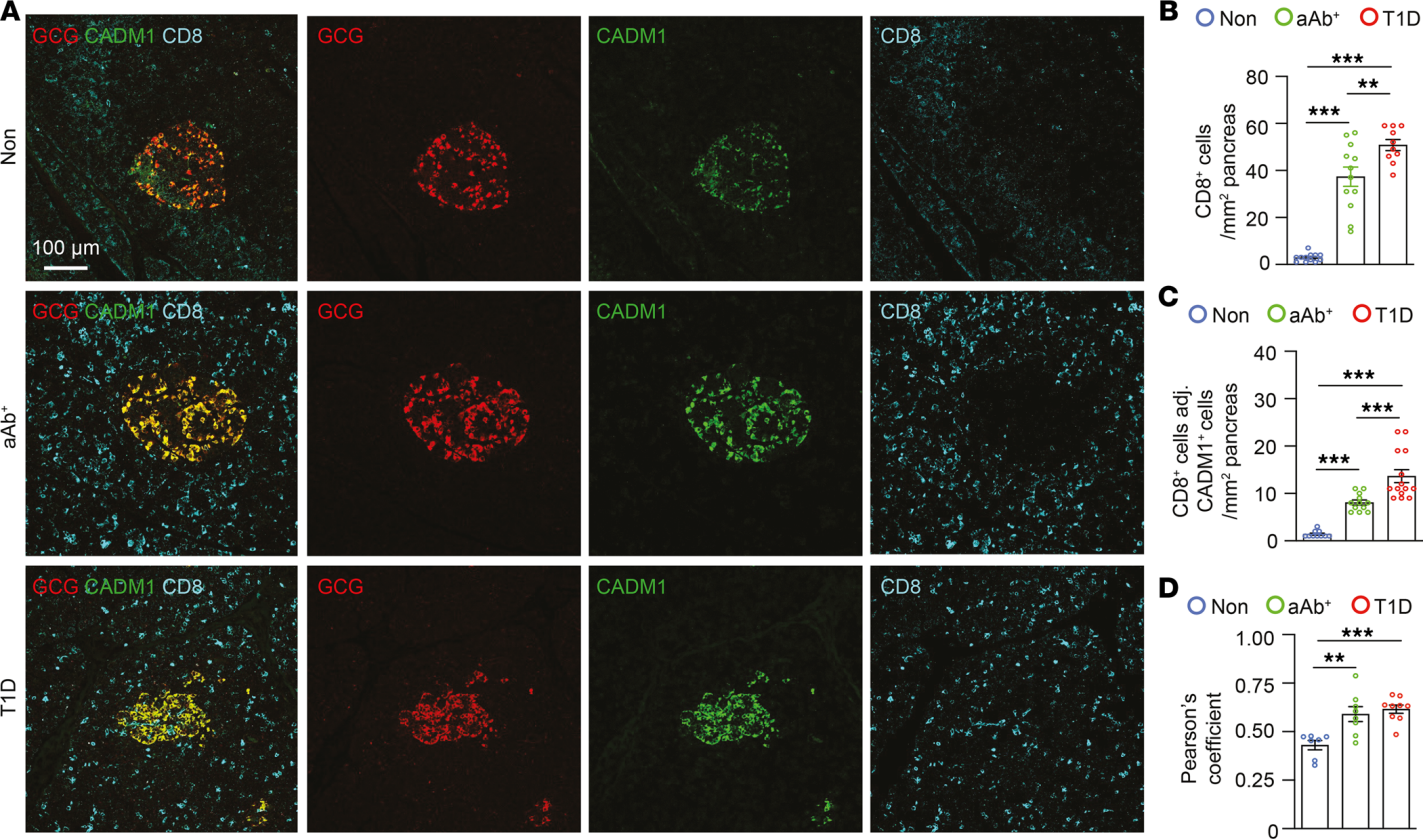
Increased number of CD8^+^ T cells adjacent to CADM1^+^GCG^+^ cells during T1D. (**A**) Immunostaining of paraffin-embedded pancreata from individuals in the Non, aAb^+^, and T1D groups for CADM1 (green), GCG (red), and CD8 (cyan). Scale bar: 100 μm. (**B**) Quantification of the number of CD8^+^ cells/mm^2^ pancreas area (*n* = 5 per group). (**C**) Quantification of the number of CD8^+^ cells adjacent to CADM1^+^ cells within the islet boundary in pancreata of individuals in the Non, aAb^+^, and T1D groups (*n* = 5 per group). (**D**) Pearson correlation indicates increased colocalization between Cadm1 and GCG expression in aAb^+^ (*n* = 5) and T1D (*n* = 5) pancreata compared with pancreata from the Non group (*n* = 5). One-way ANOVA was performed using GraphPad Prism, version 7, software for comparisons of 3 groups. Post hoc statistical analyses were performed using the Tukey multiple comparisons test. Results are presented as mean ± SEM. **P < 0.01; ***P < 0.001. adj., adjacent.

We next evaluated whether numbers of CADM1^^+^^ cells adjacent to CD8^^+^^ T cells were increased during T1D. We first performed immunostaining for CADM1^^+^^, glucagon-positive (GCG^^+^^), and CD8^^+^^ cells and quantified CD8^^+^^ cells adjacent to CADM1^^+^^ islet endocrine cells (Figure 5A). CD8^^+^^ cell numbers were increased in pancreatic sections from aAb^^+^^ individuals and those with T1D (Figure 5B), and likewise, the number of CADM1^^+^^ endocrine cells adjacent to CD8^^+^^ T cells was increased in sections from aAb^^+^^ individuals and those with T1D compared with the Non control group (Figure 5C). Colocalization of CADM1 and GCG immunostaining in pancreata from the aAb^^+^^ and T1D groups was increased compared with pancreata from the Non group, consistent with the increase in GCG^^+^^ cells (Figure 5D and Supplemental Figure 2D). These results indicate that CADM1^^+^^ islet endocrine cells may bind to the CTLs that infiltrate the pancreas during autoimmune diabetes.

**Figure 6:**
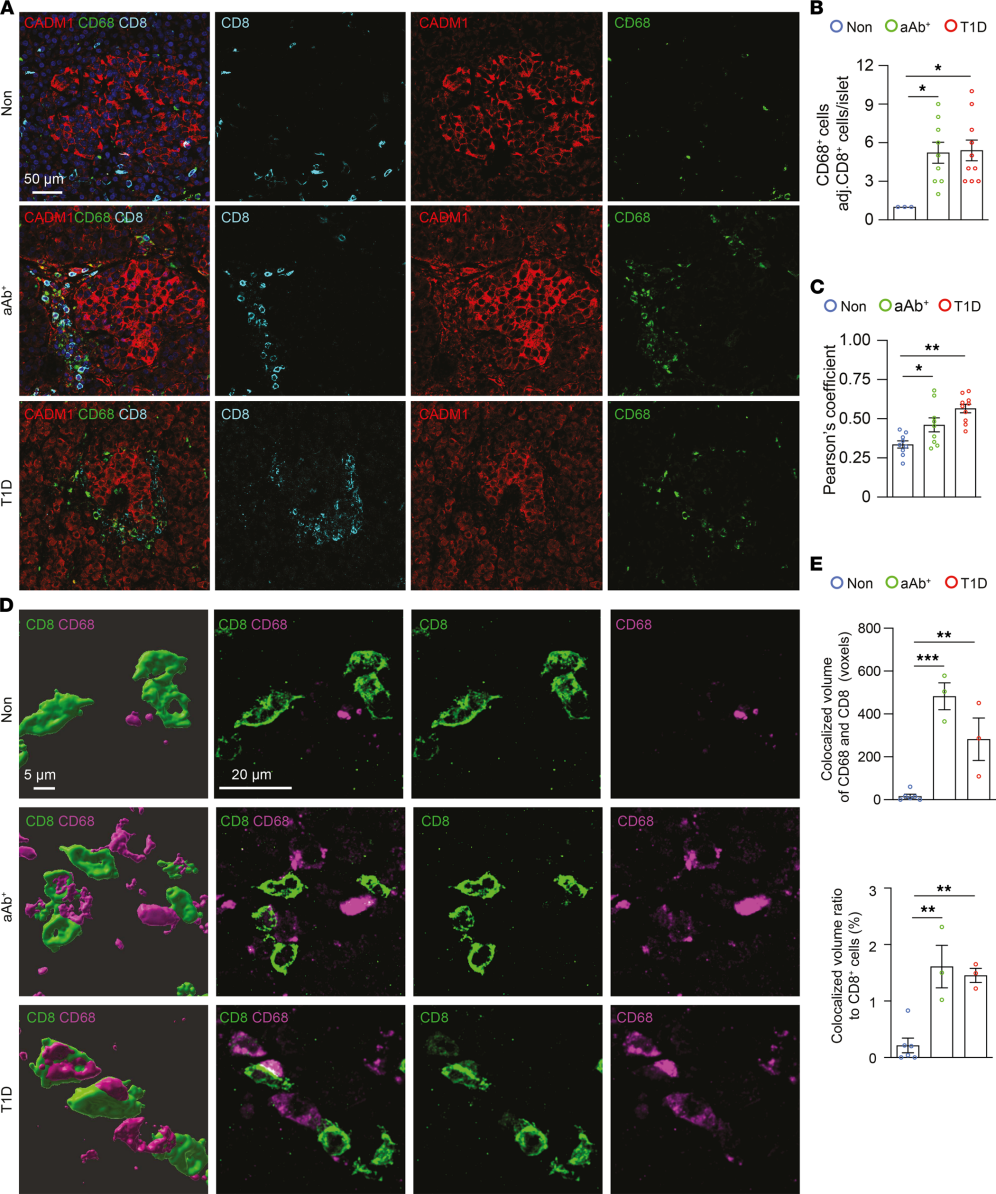
Increased number of CD8^+^ T cells adjacent to CADM1^+^CD68^+^ cells during T1D. (**A**) Immunostaining of paraffin-embedded pancreata from individuals in the Non, aAb^+^, and T1D groups for CADM1 (red), CD68 (green), and CD8 (cyan). Scale bar: 50 μm. (**B**) Quantification of the number of CADM1^+^ cells adjacent to CD8^+^ cells within the islet boundary (*n* = 5 per group). (**C**) Pearson correlation indicates increased colocalization between Cadm1 and CD68 expression in aAb^+^ (*n* = 5) and T1D (*n* = 5) pancreata compared with pancreata from the Non group (*n* = 5). (**D**) Three-dimensional rendering of immunostaining of paraffin-embedded pancreata from individuals in the Non, aAb^+^, and T1D groups for CD8 (green) and CD68 (magenta) (left column, scale bar: 5 μm). Parent images of original immunostaining (3 right-most columns, scale bar: 20 μm). (**E**) Quantification of colocalized volumes of CD68 and CD8 after immunostaining of paraffin-embedded pancreata from the Non, aAb^+^, and T1D groups for CD68 (green) and CD8 (magenta). One-way ANOVA was performed using GraphPad Prism, version 7, software for comparisons of 3 groups. Post hoc statistical analyses were performed using the Tukey multiple comparisons test. Results are presented as mean ± SEM. *P < 0.05; **P < 0.01; ***P < 0.001.

**Figure 7:**
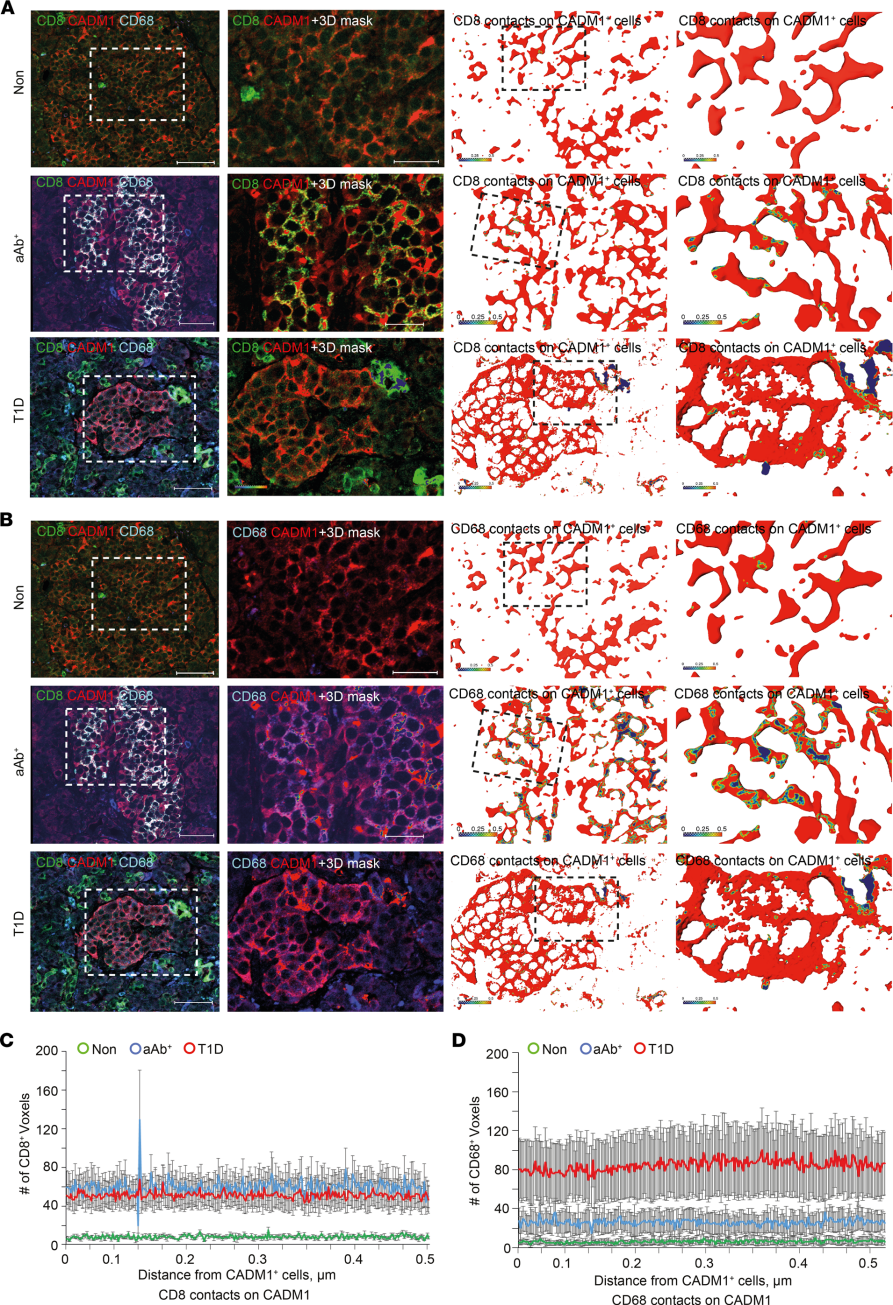
Increased colocalization of CADM1 with CD8 and CD68 during T1D. (**A** and **B**) Immunofluorescence profile (left column) and the 3D surface rendering of Amira 3D reconstruction of CADM1^+^ cells contacting CD8^+^ (**A**) and/or CD68^+^ (**B**) cells. CADM1^+^ cells are represented by the 3D reconstruction. The cell surface is indicated by red; the CADM1/CD8^+^ (**A**) and/or CADM1/CD68^+^ (**B**) contacts are color-coded, with the cool to warm colors spreading from a 0 to 500 nm distance between the surface of either CD8^+^ and/or CD68^+^ cells and the CADM1 soma (see color-coded horizontal bar for the distance definition). (**C** and **D**) Histograms showing the number of CD8-labeled and CD68-labeled voxels (3D pixels) found within 500 nm of the CADM1^+^ cell body. Two-way repeated-measure ANOVA was performed using GraphPad Prism, version 7, software. Post hoc statistical analyses were performed using the Holm-Šidák multiple comparisons test.

To test whether CADM1^^+^^CD68^^+^^ cells engage CD8^^+^^ T cells during T1D, we performed immunostaining for CADM1, CD68, and CD8 cells in the pancreatic sections from human donors (Figure 6A). Consistent with previous observations in CD45^^+^^ cells, the number of CD68^^+^^ cells were increased in both aAb^^+^^ and T1D pancreatic sections, and the number of these cells adjacent to CD8^^+^^ T cells was also significantly elevated in comparison with pancreatic sections from the Non group (Figure 4E and Figure 6B). Similarly, quantification of colocalization of CADM1 and CD68 immunostaining in pancreata from aAb^^+^^ individuals and those with T1D revealed a marked increase when compared with colocalization in pancreata from the Non group (Figure 6C). Moreover, high-resolution 3D analysis of CD8 and CD68 immunostaining showed increased colocalization of these markers in both aAb^^+^^ and T1D pancreatic sections, further suggesting increased interaction of these cell types (Figure 6, D and E). Likewise, high-resolution 3D analysis further supported (a) the increased proximity of CD8^^+^^ cells to CADM1^^+^^ cells in pancreata from the aAb^^+^^ and T1D groups compared with those from the Non group (Figure 7, A and C) as well as (b) the colocalization of CADM1^^+^^CD68^^+^^ immunoreactivity in pancreata from the aAb^^+^^ and T1D groups compared with those from the Non group (Figure 7, B and D). Together, these results demonstrate that in addition to the increased number CD68^^+^^ cells present in the islet microenvironment in aAb^^+^^ and T1D pancreata, the increased colocalization of CADM1 and CD68 can reflect either increased CADM1 expression or the increase in the number of CADM1^^+^^CD68^^+^^ cells (Figure 4E).

**Figure 8:**
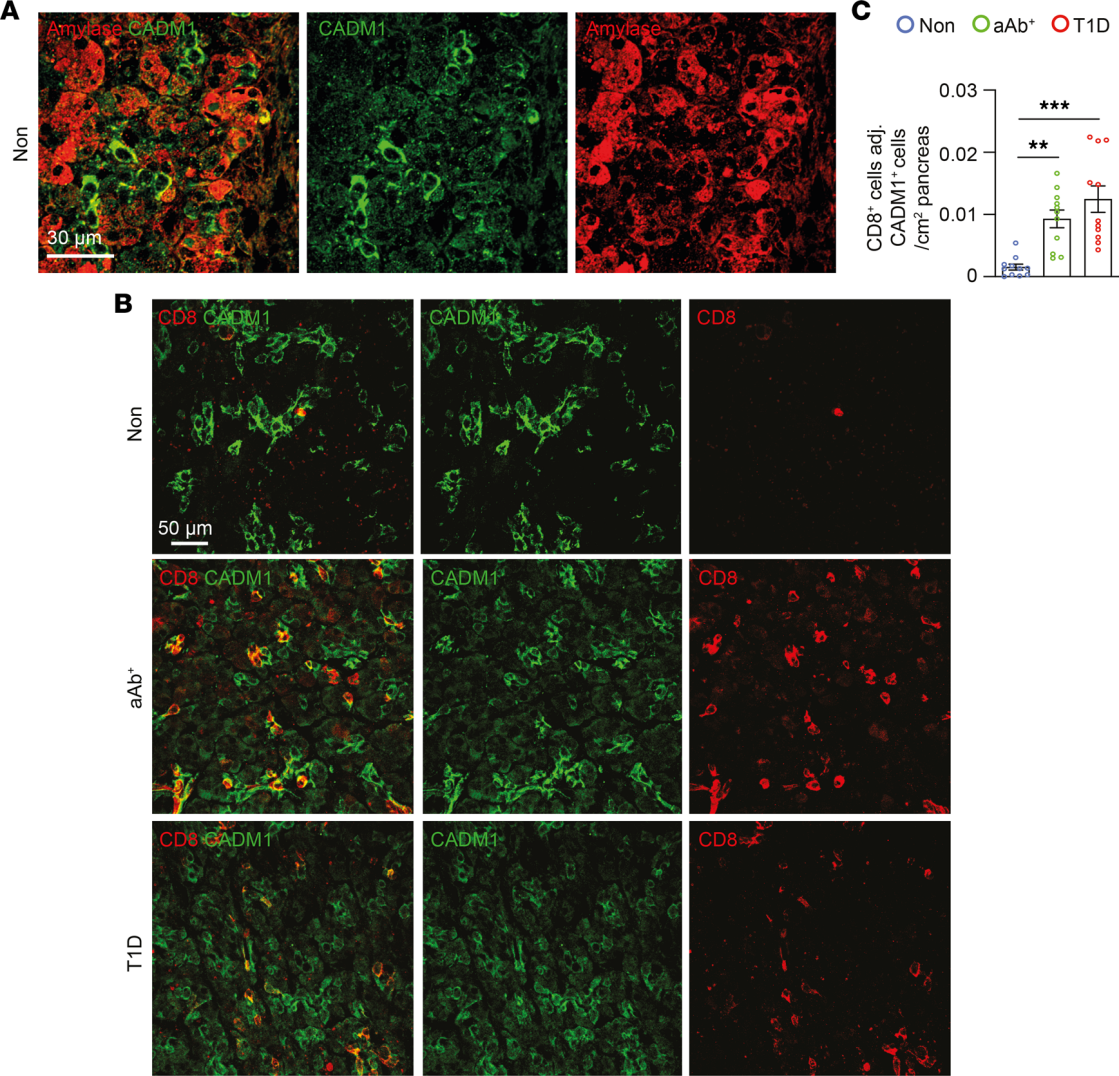
Increased number of CADM1^+^ cells adjacent to CD8^+^ T cells in the exocrine pancreas during T1D. (**A**) Immunostaining of paraffin-embedded pancreata from individuals in the Non group for CADM1 (green), and pancreatic amylase (red). Scale bar: 30 μm. (**B**) Immunostaining of paraffin-embedded pancreata from the Non, aAb^+^, and T1D groups for CADM1 (green), and CD8 (red). Scale bar: 50 μm. (**C**) Quantification of the number of CD8^+^ cells adjacent to CADM1^+^ cells/mm^2^ exocrine pancreas in pancreata of individuals in the Non, aAb^+^, and T1D groups (*n* = 5 per group). One-way ANOVA was performed using GraphPad Prism, version 7, software for comparisons of 3 groups. Post hoc statistical analyses were performed using Tukey’s multiple comparisons test. Results are presented as mean ± SEM. **P < 0.01; ***P < 0.001.

It is noteworthy that immunostaining in human pancreatic sections from the Non group identified CADM1^^+^^ cells in the exocrine pancreas, and we also observed that many of these cells expressed pancreatic amylase (Figure 8A). In addition to intercellular interactions in proximity to the pancreatic islet, the number of CADM1^^+^^ cells colocalizing with CD8^^+^^ T cells in the exocrine pancreas was increased in the aAb^^+^^ and T1D groups compared with the Non group (Figure 8, B and C). These observations collectively illustrate that CADM1 expression in multiple cell populations, including the endocrine and exocrine pancreas, may create an opportunistic environment for harboring infiltrating immune cell types, including CD8^^+^^ T cells, during the development of autoimmune diabetes in humans. The increased number of intercellular contacts between CADM1^^+^^ cell populations with CD8^^+^^ T cells indicates that inhibition of these interactions may constitute a strategy for preventing infiltration of CTLs into the pancreas and ultimately disease onset.

## Discussion

The molecular mechanisms mediating immune cell recruitment into the pancreatic islet during T1D pathogenesis remain poorly understood. Cadm1 is an established mediator of intercellular binding (20, 21), and, in our previous work, we addressed its function in the growth and function of the β cell (23, 24). The goals of the present study were to (a) analyze publicly available scRNA-Seq data sets for evidence of *Cadm1* enrichment in pancreatic immune cell populations isolated from the NOD mouse model of T1D and (b) determine whether increased CADM1-mediated cell interactions could be detected within islets of individuals with T1D.

Among the most important results of our study was identifying the enrichment of *Cadm1* in myeloid cell populations, including macrophages, isolated from pancreata of adult NOD mice. This finding is notable for several reasons. To date, Cadm1 function in myeloid cells has not been established and observing its enrichment in these cells in a model of T1D may allude to a pathogenic role for Cadm1 in instigating immune cell activation and disease progression. Consistent with this hypothesis, our results show the increased presence of CADM1^^+^^CD68^^+^^ cells adjacent to CD8^^+^^ T cells at the islet periphery in pancreata isolated from aAb^^+^^ individuals and individuals with T1D. Macrophages long have been implicated in the pathogenesis of autoimmune diabetes (31–33), and these observations identify CADM1 as a potential mediating protein in islet endocrine and myeloid populations with infiltrating cytotoxic lymphocytes. Zakharov et al. (25) highlighted that within the complex immune cell heterogeneity in islets during the development of autoimmune diabetes, resident macrophages underwent a stepwise activation program, and this sequence of events was characterized by the polarization of macrophage subpopulations. After plotting the cellular identity for each cluster identified in the scRNA-Seq analysis according to time point, the enrichment of Cadm1 in macrophages at age 8 weeks in the present study may further allude to a role for these cells in prompting the recruitment of lymphocytes that leads to the manifestation of the disease after age 15 weeks in NOD mice. In our analysis, we observed an alteration in the distribution of *Cadm1*-expressing macrophages at age 8 weeks compared with age 4 weeks, revealing the cellular heterogeneity of the macrophage population at this time point. In line with observations by Zakharov et al. (25), the increased expression of *Cadm1* at 8 weeks may indicate this adhesion molecule contributes to the polarized identity of a specific subpopulation of macrophages that facilitate immune cell recruitment and binding within the islets of the NOD model. Studies are warranted for investigating the function of Cadm1 in macrophage subtype specification and how a transient increase in its expression coincides with the recruitment of lymphoid cell types during T1D.

Interestingly, previous studies have shown that Cadm1^^+^^ cells will bind CD8^^+^^ T cells and NK cells via the receptor Class I–restricted T cell–associated molecule (Crtam) present in the immune cells (34). Crtam is an immunoglobulin-like cell surface protein, and Crtam-Cadm1 interactions have been reported to enhance NK cell and CD8^^+^^ T cell effector functions (34, 35). Disruption of Crtam-Cadm1 contact in either *Crtam* or *Cadm1* total KO mice led to a reduction of the CD4^^+^^CD8^^+^^ T cell population, and total loss of *Crtam* expression protected mice from induction of diabetes, thereby underlining the potential relevance of this interaction in mediating autoimmune destruction of pancreatic β cells (34, 35). In addition to validating CADM1 function in cell binding with the CRTAM T cell receptor, studies should be conducted to determine the significance of CADM1 expression in macrophages within an inflammatory or repair context and how it may facilitate immune cell recruitment to the endocrine pancreas.

Additional noteworthy observations made in this study were (a) the induction of CADM1 expression in the exocrine pancreas in aAb^^+^^ individuals and those with T1D and (b) the increase in the number of interactions between CADM1^^+^^ and CD8^^+^^ T cells in the exocrine pancreas. These results indicate CADM1 expression in acinar cells appears to increase in the presence of autoantibodies and, together with our observations in CD68^^+^^ myeloid cells, these observations suggest multiple CADM1-expressing cell populations could potentially mediate contact to CTLs prior to manifestation of T1D. Although expression of CADM1 in the endocrine pancreas appears stable, on the basis of immunostaining results, the induction of CADM1 in exocrine cells may identify a previously undescribed role for these cells in mediating immune cell recruitment. Further investigation is necessary to determine whether these CADM1-mediated interactions in the exocrine pancreas precede infiltration of the islet and/or cytotoxic destruction of β cells.

In our previous work, we identified *Cadm1* as a direct target of miRNA 375 (miR-375), the most abundant miRNA in the pancreatic β cell (22, 36). Although the precise role of the miRNA pathway is not clear, based on our observations presented here, we also hypothesize that a potential function of miR-375 in its direct suppression of *Cadm1* may be to prevent immune cell infiltration into the islet microenvironment. Notably, both miR-375 and *Cadm1* are also expressed in pancreatic α cells (22, 36), and although this functional interaction has not been validated in this cell type, our result showing increased colocalization of CADM1 and GCG in aAb^^+^^ and T1D pancreata may also allude to an important role for miR-375 in restricting CADM1 expression in the α cell compartment to minimize interaction with infiltrating immune cells.

Furthermore, recent genome-wide association studies have identified an association between BMI and a locus near *CADM1* (37), and we showed that this BMI risk variant (single nucleotide polymorphism rs12286929) correlates with increased *CADM1* expression in multiple brain regions of humans (38). In addition, an increased association of obesity and T1D has also been reported (39–41); together, these findings may begin to suggest a possible link connecting alterations in *CADM1* expression with increased body weight and the progression of autoimmune diabetes. Consistent with this hypothesis, in the present investigation, the overall mean BMI of individuals in the T1D cohort was higher than in the Non group; however, further investigation is necessary to determine whether this BMI risk variant is associated with T1D susceptibility.

Administration of a monoclonal Ab against the marker CD11b into NOD mice prevented intra-islet infiltration of macrophages as well as β cell death and hyperglycemia (31). More recently, researchers have begun to explore the contribution of neuronal and immune cell interactions to metabolic dysfunction in peripheral tissues, including the pancreas (42). Islet-resident macrophages are in close proximity to islet nerve fibers, and both sympathetic denervation and α-1 adrenergic receptor inhibition halted the aggressive immune response in the pancreas after induction of autoimmune diabetes in mice (43). These observations support the hypothesis that immune cell and islet cell contact contributes to autoimmune destruction of the pancreas (44); however, the factors mediating cell-cell contact with islet macrophages have not been established.

In summary, the precise function of CADM1 in myeloid cells present in the islet microenvironment during diabetes pathogenesis had not been studied before, to our knowledge, and here we report its identification as a mediator of immune cell interactions in this context. To date, little is known regarding (a) the factors that define islet macrophage populations, (b) how these factors mediate immune cell recruitment into the islet, or (c) how these factors contribute to β cell dysfunction and failure. Here we show that increased numbers of CD68^^+^^ myeloid cells are CADM1^^+^^ in pancreata of aAb^^+^^ individuals and of individuals with T1D, compared with the pancreata of individuals without diabetes, and these cells are adjacent to CD8^^+^^ T cells, suggesting that induction of CADM1 in macrophage or DC populations may contribute to the pathogenesis of autoimmune diabetes. These results also indicate that as a membrane protein, strategies in the form of blocking Abs raised against the extracellular portion of CADM1 may prevent engagement with infiltrating T cells and constitute a viable therapeutic approach for preventing β cell destruction and disease onset. Studies will be conducted to pursue to these goals using both genetic and pharmacologic approaches to block Cadm1-mediated intercellular binding and to test its direct role in T1D pathogenesis.

## Methods

### Human donor samples

Human pancreatic tissue samples were obtained from the nPOD biorepository at the University of Florida in Gainesville, Florida (https://www.jdrfnpod.org). Tissue samples were selected that met the following specific criteria: (a) to minimize variance in age across all individuals, and (b) duration of diabetes for individuals with T1D was kept as brief as possible. Paraffin-embedded tissue samples were from 3 donor groups: individuals with T1D (*n* = 5), aAb^^+^^ individuals (*n* = 5), and the Non control group (*n* = 5). The following information is documented in Supplemental Table 1: nPOD case identification number, autoantibody status, age, duration of diabetes, sex, race, BMI, HbA1c and C-peptide levels, and cause of death. Samples were recovered following standard operating procedures, including receipt informed research consent by organ procurement organizations throughout the US transplantation network, and shipped to the nPOD Organ Processing and Pathology Core (OPPC) at the University of Florida, as described (45).

### Analytic procedures

#### Abs for IHC analysis and cell quantification.

The following primary Abs were used for immunostaining: anti-CADM1 (clone 3E1, MBL CM004-3), anti-insulin (Invitrogen, catalog PA1-26938), anti-glucagon (Millipore, catalog MABN238), anti-CD8 (Abcam, catalog ab27605), anti-CD68 (Thermo Fisher Scientific, catalog 60291-1-IG), anti-CD11c (ICRF3.9; Abcam, catalog ab52632), and anti-CD45 (Proteintech, catalog 20103-1-AP).

#### scRNA-Seq data analysis.

Single-cell gene expression barcode, feature, and count matrices were downloaded from the GSE141784 superseries (Gene Expression Omnibus). Downstream analysis was carried out with R, version 4.1.0 (R Foundation). Quality control, filtering, data clustering, marker gene analysis, and visualization were carried out using the Seurat (version 4.0.3) R package with some custom modifications to the standard pipeline (discussed later in Methods) (46). Genes expressed in fewer than 5 cells and cells with a number of detected genes within the lower quantile (quantile 0.975) were removed from the gene expression matrix (Supplemental Figure 1A). We removed any single cell with greater than 5% unique molecular identifiers mapped to mitochondrial genes (Supplemental Figure 1B), as well as outliers with unique molecular identifier counts in the upper quantile (quantile 97.5) (Supplemental Figure 1C).

After log-normalizing the data, the expression of each gene was scaled, and principal component analysis was performed on the top 1000 most variable genes. Data integration was carried out using the Harmony algorithm (47). Harmony embeddings were used for dimension reduction, clustering, and visualization. Unsupervised shared nearest neighbor clustering was performed with a k parameter of 60 using the Leiden algorithm, and visualization was done using UMAP. Cell type annotations were predicted using the *SingleR* package and the ImmGen database as a reference (26, 27). Cluster marker genes were identified using the Wilcoxon test only considering genes with a *P* value less than 0.05, change greater than log__2__(0.25) fold change, and expressed in at least 20% of cells in the cluster. Feature plots were generated using the *Nebulosa* package (48). To infer, explore, and visualize cell-cell communication patterns, we used CellChat with default parameters (28).

#### High-resolution 3D analysis.

Samples were scanned using a Plan-Apochromat ×63/1.32 oil differential interference contrast objective at a resolution of 1024 × 1024 pixels with 8-bit sampling in sequential scanning frame-by-frame mode. Single optical sections were acquired using identical acquisition settings, with the pinhole of 1 Airy unit. Stacks of 8 to 29 optical sections yielded voxel dimensions between 100 and 400 nm for the *x*, *y*, and *z* planes. Three-dimensional reconstructions were generated with Amira Software 2020.2 (Thermo Fisher Scientific). First, the surface area of the CADM1^^+^^, CD8^^+^^, and/or CD68^^+^^ cells was reconstructed using the Amira segmentation editor. CADM1/CD8^^+^^ and/or CADM1/CD68^^+^^ contacts were defined by color-coding the surface of CD8^^+^^ and/or CD68^^+^^ cells found within 500 nm of CADM1^^+^^ soma. Subsequently, the surface of 500 nm distant CD8^^+^^ and/or CD68^^+^^ voxels was mapped onto CADM1^^+^^ cells using the surface-distance tool and plotted as a histogram. For 3D colocalization analysis, 3D imaging reconstructions were performed with Imaris software (version 9.8; Oxford Instruments) using the Imaris surface editor. Colocalization was analyzed with ImarisColoc plugins.

#### Data availability.

The scRNA-Seq data used in this study are publicly available in the Gene Expression Omnibus under accession number GSE141786 (25).

### Statistics

All results are expressed as mean ± SEM, and the statistical analyses are summarized in Supplemental Table 2. Comparisons between data sets with 2 groups were evaluated using an unpaired 2-tailed Student’s *t* test. One-way and 2-way repeated-measures ANOVA was performed using GraphPad Prism, version 7, software for comparisons of 3 or more groups. Post hoc statistics were performed using Tukey’s or Holm-Šidák multiple comparison test. *P* ≤ 0.05 was considered statistically significant. The presented data met the assumptions of the statistical tests used. Normality and equal variances were tested using GraphPad Prism software. No statistical methods were used to predetermine sample sizes, but our sample sizes were similar to those reported in previous publications.

### Study approval

All experiments using nPOD human donor tissue were conducted with the approval of the Johns Hopkins University Institutional Review Board (IRB00244487). For preparation of human tissue, all procedures were performed according to the established standard operating procedures of the nPOD/OPPC and approved by the University of Florida Institutional Review Board (IRB201600029) and the United Network for Organ Sharing according to federal guidelines, with written informed consent obtained from each donor’s legal representative.

## Author contributions

CS, YTY, and NLK performed the IHC and morphometric analysis. AP, LH, and LN reanalyzed the single-cell sequencing and processed the data using computational workflows. XY performed statistical analysis. MNP conceived and designed the study and wrote the manuscript. All authors contributed to the interpretation of the data and approved the final version of this manuscript. Order of first authors was determined by (a) time dedicated to conducting experiments that resulted in published figures and (b) the amount of conceptual input to the manuscript and overall study.

## Supplementary Material

Supplemental data

ICMJE disclosure forms

## Version 1 02/08/2022

In-Press Preview

## Version 2 03/22/2022

Electronic publication
