# Supplementary material for: Evidence of islet CADM1-mediated immune cell interactions during human type 1 diabetes
Source: JCI Insight. 2022 Mar 22;7(6):e153136. doi: 10.1172/jci.insight.153136 (PMC8986082; doi:10.1172/jci.insight.153136)
Supplement: Supplemental data [file jciinsight-7-153136-s207.pdf]

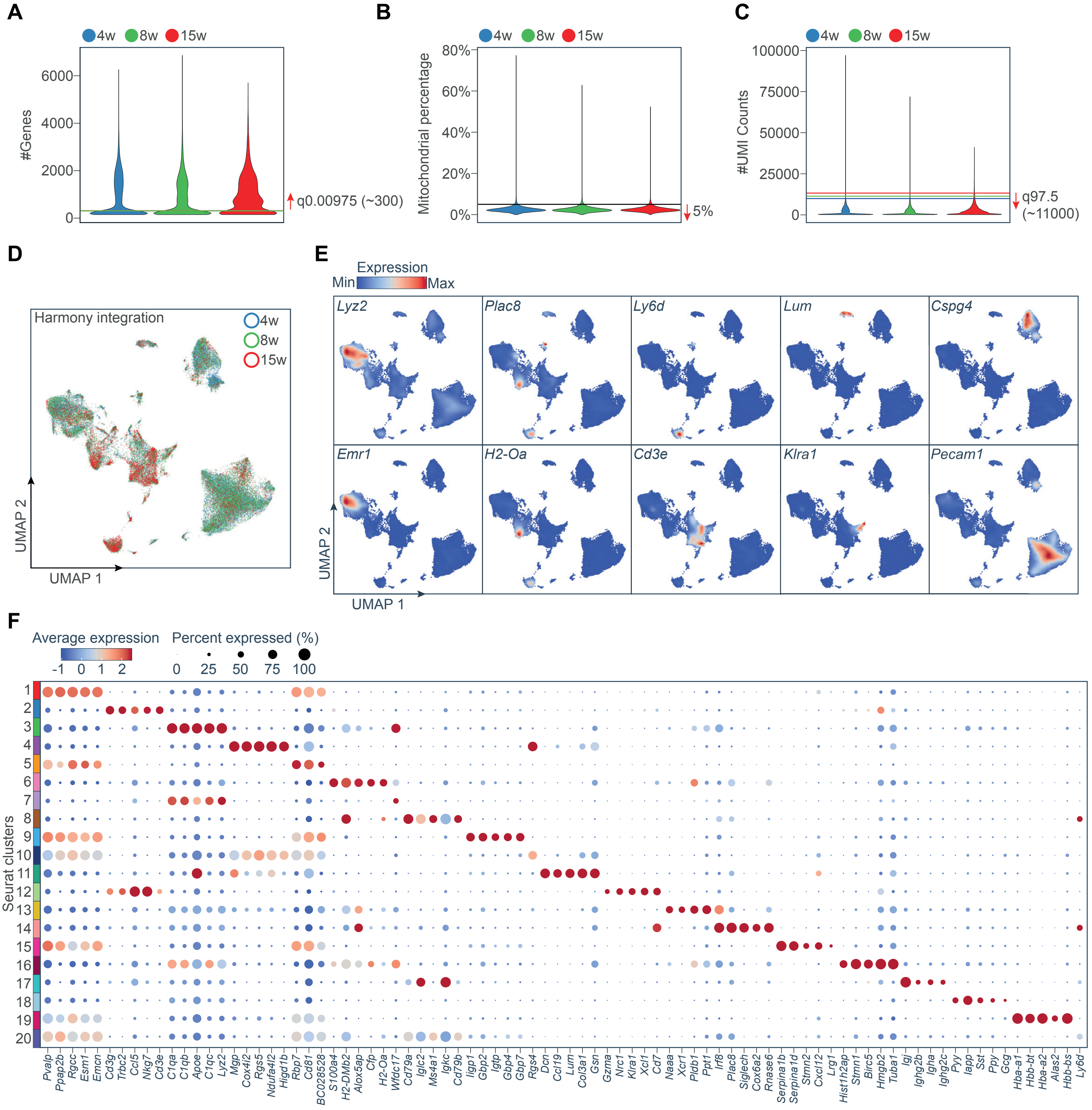

Supplemental Figure 1. Single-cell analysis revealed novel markers in islet-infiltrating cells during diabetes development. Violin plots representing the (**A**) number of genes per cell, (**B**) mitochondrial genes percentage of all cells before QC, and (**C**) number of unique molecular identifiers (UMIs) before filtering and clustering. Colored lines indicate the filtering parameter values set for each sample (q indicates quartile). (**D**) Harmony integration of pancreatic islet cells pooled together from 4, 8, and 15-week-old NOD mice. (**E**) Feature plots of expression distribution for selected cluster-specific genes. Expression levels for each cell are color-coded and overlaid onto UMAP plot. (**F**) Top 5 marker genes for the 20 identified clusters. The dot size represents the percentage of cells within a group with an expression level > 0 and color-scale represents the average expression level (row Z-score) across all cells within the cluster.

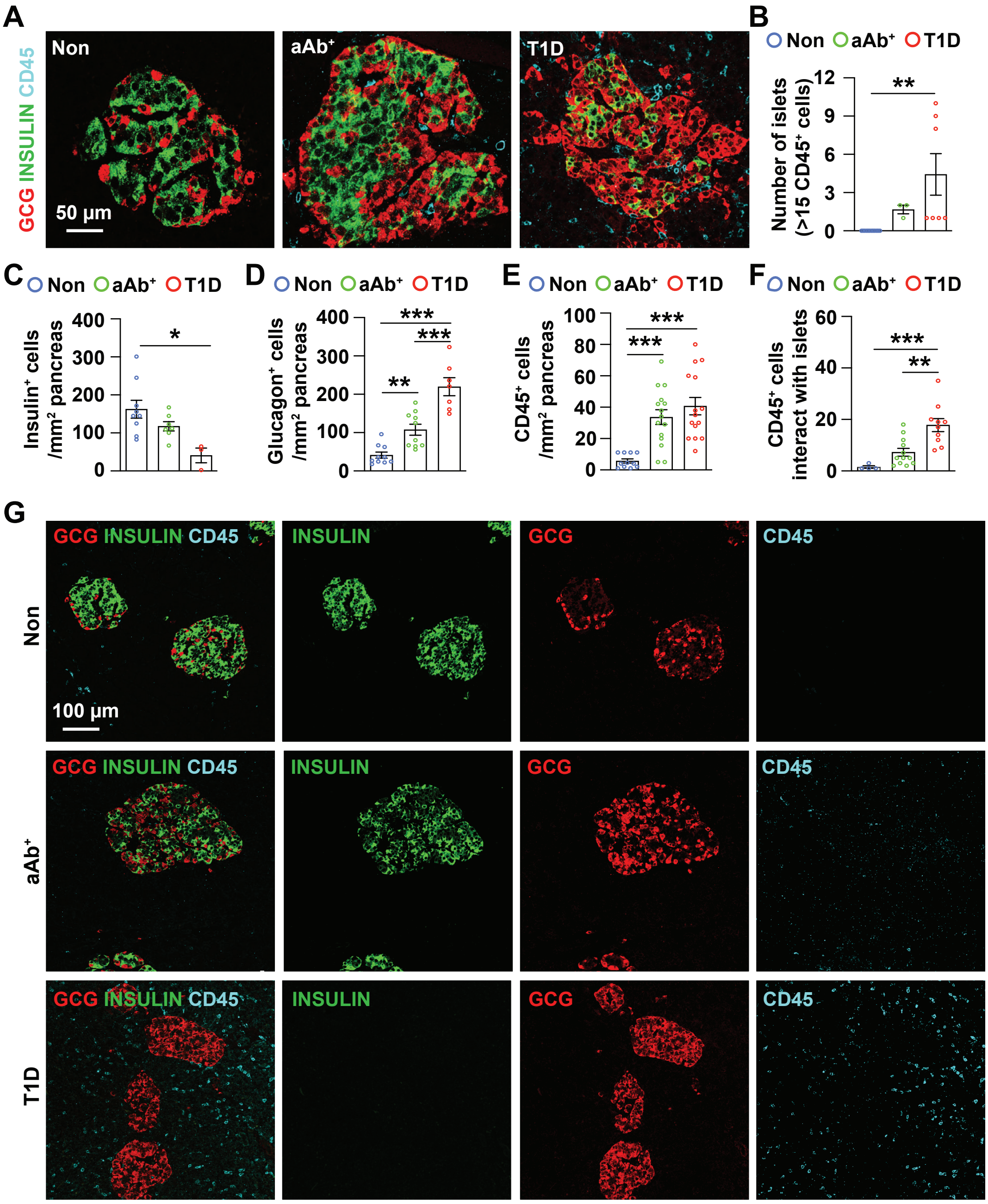

Supplemental Figure 2. Control immunostaining for human pancreata from nPOD repository. (**A**) Immunostaining of paraffin-embedded pancreata from non-diabetic (Non), auto-antibody positive (aAb$^+$), and T1D human subjects for insulin (green), glucagon (red), and CD45 (cyan). Scale bar: 50 µm. (**B**) Quantification of number of islets with >15 CD45$^+$ cells within the islet periphery per mm^2 pancreas from Non, aAb$^+$, and T1D human subjects. (**C**) Quantification of number of insulin$^+$ cells per mm^2 pancreas from Non, aAb$^+$, and T1D human subjects. (**D**) Quantification of number of glucagon$^+$ cells per mm^2 pancreas from Non, aAb$^+$, and T1D human subjects. (**E**) Quantification of number of CD45$^+$ cells per mm^2 pancreas from Non, aAb$^+$, and T1D human subjects. (**F**) Quantification of number of CD45$^+$ cells adjacent to insulin$^+$ or glucagon$^+$ cells in pancreata of Non, aAb$^+$, and T1D human subjects. (**G**) Representative images of immunostaining of glucagon (GCG) (red), insulin (green), and CD45 (cyan) in pancreata of Non, aAb$^+$, and T1D human subjects. Scale bar: 100 µm. One-way ANOVA analysis has been performed using GraphPad Prism Software Version 7 for comparisons of three groups. Post hoc statistics were performed using Turkey's multiple comparisons test. Results are presented as mean ± S.E.M. *P< 0.05, **P< 0.01, ***P< 0.001.

Sona et al., 2021
Supplemental Figure 2

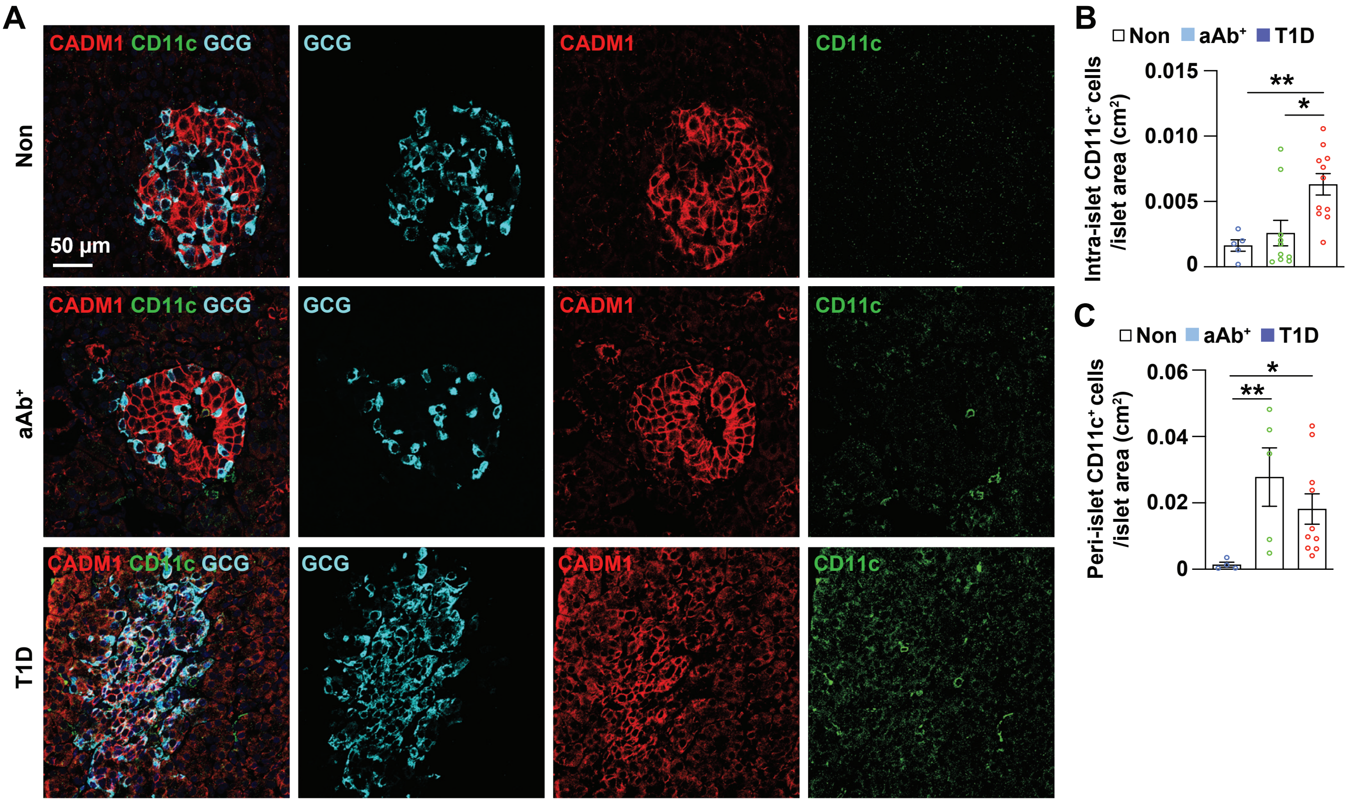

Supplementary Figure 3. Increased number of CADM1^{+} cells adjacent to CD11c^{+} cells during T1D. (**A**) Immunostaining of paraffin-embedded pancreata from non-diabetic (Non), auto-antibody positive (aAb^{+}), and T1D human subjects for CADM1 (red), glucagon (cyan) and CD11c (green). Scale bar: 50 µm. (**B**) Quantification of number of CD11c^{+} cells within the islet boundary per cm^2 endocrine pancreas of Non, aAb^{+}, and T1D human subjects. (**C**) Quantification of number of CD11c^{+} cells at the islet periphery per cm^2 endocrine pancreas of Non, aAb^{+}, and T1D human subjects. One-way ANOVA analysis has been performed using GraphPad Prism Software Version 7 for comparisons of three groups. Post hoc statistics were performed using Turkey's multiple comparisons test. Results are presented as mean ± S.E.M. **P*< 0.05, ***P*< 0.01, ****P*< 0.001.

Supplemental Table 1. Demographic information of human pancreatic tissue donors from nPOD program and summary of available donor data including auto-antibody results: GADA, glutamic acid decarboxylase-65; IA-2A, islet antigen-2 or insulinoma antigen-2; ZnT8A, zinc transporter 8; mIAA, insulin autoantibodies. Insulin autoantibody (mIAA) is an important marker of islet autoimmunity and is utilized in confirmation of type 1 diabetes diagnosis; however, assay specificity after a person has been on exogenous insulin for more than 10 days, is no longer able to distinguish insulin autoantibodies from insulin antibodies which form in over 40% of patients treated with subcutaneous insulin (Fineberg et al. *Diabetologia* (1983)). Therefore, mIAA is not a reliable marker of autoimmunity after a donor has been on insulin therapy for more than 10 days.

nPOD CaseID	RRID	Donor Type ID	Auto-antibody_Results	Age (yr)	Duration of diabetes (yrs)	Gender	Race	BMI (kg/m2)	HbA1c	C-peptide	Cause of death
6310	SAMN15879364	Autoab Pos	GADA+	28	-	Female	Hispanic/Latino	22.4	n/a	10.54	Anoxia
6314	SAMN15879368	Autoab Pos	GADA+	21	-	Male	Caucasian	23.8	n/a	1.49	Head Trauma
6424	SAMN15879477	Autoab Pos	GADA+, mIAA+	17.65	-	Male	Caucasian	51.4	5.8	6.97	Head Trauma
6429	SAMN15879482	Autoab Pos	GADA+, mIAA+	22.1	-	Male	African Am	19.6	5.5	2.25	Head Trauma
6450	SAMN15879503	Autoab Pos	GADA+, ZnT8A+	22	-	Female	Caucasian	24.4	5.7	5.47	Anoxia
6512	SAMN15879564	Autoab Pos	IA2A+, mIAA+, ZnT8A+	30.59	-	Female	Caucasian	38.8	5.2	3.2	Cerebrovasc./Stroke
6517	SAMN18053201	Autoab Pos	GADA+	22.14	-	Male	Caucasian	26.6	5.6	9.74	Cerebrovasc./Stroke
6521	SAMN18053204	Autoab Pos	GADA+, IA-2A+, ZnT8A+	19.77	-	Male	Hispanic/Latino	24.1	5.8	7.44	Head Trauma
6148	SAMN15879204	T1D	GADA+, mIAA+	17.1	7	Male	Caucasian	23.9	n/a	<0.05	Anoxia
6195	SAMN15879251	T1D	GADA+, Ia-2A+, mIAA+, ZnT8A+	19.3	5	Male	Caucasian	23.7	n/a	<0.05	Head Trauma
6480	SAMN15879533	T1D	IA2A+, mIAA+	17.18	2.5	Male	Caucasian	27.0	10.2	0.13	Diabetic ketoacidosis
6526	SAMN18053209	T1D	IA2A+, mIAA+	29.74	1	Male	Hispanic/Latino	23	6.6	0.07	Anoxia
6524	SAMN18053207	T1D	GADA+, Ia-2A+, mIAA+, ZnT8A+	20.07	8	Male	Caucasian	29.5	6.5	0.03	Head Trauma
6520	SAMN18053203	T1D	GADA+, Ia-2A+, ZnT8A+	21.61	0	Male	Caucasian	29.3	11.9	0.37	Cerebrovasc./Stroke
6449	SAMN15879502	T1D	IA-2A+, mIAA+, ZnT8A+	24	2	Male	Caucasian	23	10.2	0.03	Head Trauma
6399	SAMN15879452	T1D	GADA+, IA-2A+, ZnT8A+	17.42	0	Male	Caucasian	32	10.4	1.41	Anoxia
6098	SAMN15879155	No diabetes	negative	17.8	-	Male	Caucasian	22.8	4.9	1.41	Head Trauma
6174	SAMN15879230	No diabetes	negative	20.9	-	Male	Caucasian	19.5	n/a	3	Cerebrovasc./Stroke
6389	SAMN15879442	No diabetes	negative	18.6	-	Male	Caucasian	20.93	5.1	7.22	Head Trauma
6516	SAMN18053200	No diabetes	negative	20.75	-	Male	Caucasian	28.8	5.5	8.91	Head Trauma
6492	SAMN15879545	No diabetes	negative	28.56	-	Male	Hispanic/Latino	26.6	6.2	2.13	Head Trauma
6509	SAMN15879561	No diabetes	negative	18.72	-	Male	Caucasian	25.8	5.4	2.21	Head Trauma
6479	SAMN15879532	No diabetes	negative	21.67	-	Female	Hispanic/Latino	20.9	5	1.74	Anoxia
6229	SAMN15879285	No diabetes	negative	31	-	Female	Caucasian	26.9	5.5	6.23	Head Trauma

Supplemental Table 2. Summary of statistical analyses. All statistical analysis for each figure including n numbers for each test cohort, statistical test performed, and resulting P values for all comparisons.

Figure	Sample size (n)	Statistical Test	Values
**3C**	ND (n=7)<br>aAb+ (n=9)<br>T1D (n=10)	Ordinary one-way ANOVA<br>Turkey's multiple comparisons test	Summary: F=3.602, P =0.0432<br>Multiple comparison:<br>ND vs aAb^+: P=0.3466<br>ND vs T1D: P=0.0344<br>T1D vs aAb^+: P=0.4014
**3D**	ND (n=14)<br>aAb+ (n=12)<br>T1D (n=11)	Ordinary one-way ANOVA<br>Turkey's multiple comparisons test	Summary: F=40.55, P<0.0001<br>Multiple comparison:<br>ND vs aAb^+: P=0.0002<br>ND vs T1D: P<0.0001<br>T1D vs aAb^+: P=0.0004
**4B**	ND (n=17)<br>aAb+ (n=15)<br>T1D (n=10)	Ordinary one-way ANOVA<br>Turkey's multiple comparisons test	Summary: F = 15.07, P<0.0001<br>Multiple comparison:<br>ND vs aAb^+: P=0.0026<br>ND vs T1D: P<0.0001<br>T1D vs aAb^+: P=0.1168
**4C**	ND (n=17)<br>aAb+ (n=15)<br>T1D (n=10)	Ordinary one-way ANOVA<br>Turkey's multiple comparisons test	Summary: F=16.01, P<0.0001<br>Multiple comparison:<br>ND vs aAb^+: P<0.0001<br>ND vs T1D: P=0.0001<br>T1D vs aAb^+: P=0.9808
**4D**	ND (n=13)<br>aAb+ (n=14)<br>T1D (n=10)	Ordinary one-way ANOVA<br>Turkey's multiple comparisons test	Summary: F=12.78, P<0.0001<br>Multiple comparison:<br>ND vs aAb^+: P=0.1074<br>ND vs T1D: P<0.0001<br>T1D vs aAb^+: P=0.0085
**4E**	ND (n=7)<br>aAb+ (n=6)<br>T1D (n=7)	Ordinary one-way ANOVA<br>Turkey's multiple comparisons test	Summary: F=12.44, P=0.0005<br>Multiple comparison:<br>ND vs aAb^+: P=0.048<br>ND vs T1D: P=0.0003<br>T1D vs aAb^+: P=0.0996
**5B**	ND (n=12)<br>aAb+ (n=12)<br>T1D (n=10)	Ordinary one-way ANOVA<br>Turkey's multiple comparisons test	Summary: F=77.35, P<0.0001<br>Multiple comparison:<br>ND vs aAb^+: P<0.0001<br>ND vs T1D: P<0.0001<br>T1D vs aAb^+: P=0.0061
**5C**	ND (n=10)<br>aAb+ (n=12)<br>T1D (n=14)	Ordinary one-way ANOVA<br>Turkey's multiple comparisons test	Summary: F=37.79, P<0.0001<br>Multiple comparison:<br>ND vs aAb^+: P=0.0002<br>ND vs T1D: P<0.0001<br>T1D vs aAb^+: P=0.0006
**5D**	ND (n=7)<br>aAb+ (n=8)<br>T1D (n=9)	Ordinary one-way ANOVA<br>Turkey's multiple comparisons test	Summary: F=11.38, P=0.0004<br>Multiple comparison:<br>ND vs aAb^+: P=0.003<br>ND vs T1D: P=0.0005<br>T1D vs aAb^+: P=0.7949
**6B**	ND (n=3)<br>aAb+ (n=10)<br>T1D (n=11)	Ordinary one-way ANOVA<br>Turkey's multiple comparisons test	Summary: F=4.340, P=0.028<br>Multiple comparison:<br>ND vs aAb^+: P=0.0375<br>ND vs T1D: P=0.0275<br>T1D vs aAb^+: P=0.9853
**6C**	ND (n=9)<br>aAb+ (n=9)<br>T1D (n=9)	Ordinary one-way ANOVA<br>Turkey's multiple comparisons test	Summary: F=12.64, P=0.0002<br>Multiple comparison:<br>ND vs aAb^+: P=0.034<br>ND vs T1D: P=0.0001<br>T1D vs aAb^+: P=0.0762
**6E**	ND (n=6)<br>aAb+ (n=3)<br>T1D (n=3)	Ordinary one-way ANOVA<br>Turkey's multiple comparisons test	Summary: F=24.62, P=0.0002<br>Multiple comparison:<br>ND vs aAb^+: P=0.0002<br>ND vs T1D: P=0.0094<br>T1D vs aAb^+: P=0.0748
**6F**	ND (n=6)<br>aAb+ (n=3)<br>T1D (n=3)	Ordinary one-way ANOVA<br>Turkey's multiple comparisons test	Summary: F=16.32, P=0.001<br>Multiple comparison:<br>ND vs aAb^+: P=0.0021<br>ND vs T1D: P=0.0046<br>T1D vs aAb^+: P=0.8829

**7C**	ND (n=5)<br>aAb+ (n=5)<br>T1D (n=5)	Two-way repeated-measure ANOVA<br>Post-hoc multiple comparisons test (Sidak's)	Interaction: F = 0.1505, P>0.9999<br>Distance: F = 0.1843, P>0.9999<br>Genotype: F =1310, P<0.0001
**7D**	ND (n=5)<br>aAb+ (n=5)<br>T1D (n=5)	Two-way repeated-measure ANOVA<br>Post-hoc multiple comparisons test (Sidak's)	Interaction: F = 0.03389, P>0.9999<br>Distance: F = 0.03359, P>0.9999<br>Genotype: F =983, P<0.0001
**8C**	ND (n=10)<br>aAb+ (n=11)<br>T1D (n=11)	Ordinary one-way ANOVA<br>Turkey's multiple comparisons test	Summary: F=12.45, P=0.0001<br>Multiple comparison:<br>ND vs aAb^+: P=0.0049<br>ND vs T1D: P=0.0001<br>T1D vs aAb^+: P=0.3192
**Suppl.2B**	ND (n=12)<br>aAb+ (n=3)<br>T1D (n=7)	Ordinary one-way ANOVA<br>Turkey's multiple comparisons test	summary: F = 7.330, P = 0.0044<br>Multiple comparison:<br>ND vs aAb^+: P=0.5485<br>ND vs T1D: P=0.0031<br>T1D vs aAb^+: P=0.2516
**Suppl.2C**	ND (n=9)<br>aAb+ (n=7)<br>T1D (n=3)	Ordinary one-way ANOVA<br>Turkey's multiple comparisons test	Summary: F=5.686, P=0.0136<br>Multiple comparison:<br>ND vs aAb^+: P=0.2636<br>ND vs T1D: P=0.0113<br>T1D vs aAb^+: P=0.1382
**Suppl.2D**	ND (n=10)<br>aAb+ (n=10)<br>T1D (n=7)	Ordinary one-way ANOVA<br>Turkey's multiple comparisons test	Summary: F=33.11, P<0.0001<br>Multiple comparison:<br>ND vs aAb^+: P=0.0075<br>ND vs T1D: P<0.0001<br>T1D vs aAb^+: P<0.0001
**Suppl.2E**	ND (n=11)<br>aAb+ (n=15)<br>T1D (n=15)	Ordinary one-way ANOVA<br>Turkey's multiple comparisons test	Summary: F=13.95, P<0.0001<br>Multiple comparison:<br>ND vs aAb^+: P=0.2665<br>ND vs T1D: P=0.0006<br>T1D vs aAb^+: P=0.0018
**Suppl.2F**	ND (n=4)<br>aAb+ (n=12)<br>T1D (n=10)	Ordinary one-way ANOVA<br>Turkey's multiple comparisons test	Summary: F=12.67, P=0.0002<br>Multiple comparison:<br>ND vs aAb^+: P=0.0007<br>ND vs T1D: P<0.0001<br>T1D vs aAb^+: P=5128
**Suppl.3B**	ND (n=5)<br>aAb+ (n=10)<br>T1D (n=11)	Ordinary one-way ANOVA<br>Turkey's multiple comparisons test	Summary: F=7.513, P=0.0031<br>Multiple comparison:<br>ND vs aAb^+: P=0.7957<br>ND vs T1D: P=0095<br>T1D vs aAb^+: P=0.0106
**Suppl.3C**	ND (n=4)<br>aAb+ (n=5)<br>T1D (n=10)	Ordinary one-way ANOVA<br>Turkey's multiple comparisons test	Summary: F=3.644, P=0.0497<br>Multiple comparison:<br>ND vs aAb^+: P=0.0411<br>ND vs T1D: P=0.1618<br>T1D vs aAb^+: P=0.4723
**Suppl.Table 1**	ND (n=8)<br>aAb+ (n=8)<br>T1D (n=8)	Two-tailed unpaired Student's t-tes	ND vs aAb^+: t=1.243, P=0.2344<br>ND vs T1D: t=1.426, P=0.1758<br>T1D vs aAb^+: t=0.6131, P=0.5496
